# KSHV MicroRNAs Repress Tropomyosin 1 and Increase Anchorage-Independent Growth and Endothelial Tube Formation

**DOI:** 10.1371/journal.pone.0135560

**Published:** 2015-08-11

**Authors:** Philippe Kieffer-Kwon, Christine Happel, Thomas S. Uldrick, Dhivya Ramalingam, Joseph M. Ziegelbauer

**Affiliations:** HIV and AIDS Malignancy Branch, National Cancer Institute, National Institutes of Health, Bethesda, Maryland, United States of America; National University of Singapore, SINGAPORE

## Abstract

Kaposi’s sarcoma (KS) is characterized by highly vascularized spindle-cell tumors induced after infection of endothelial cells by Kaposi’s sarcoma-associated herpesvirus (KSHV). In KS tumors, KSHV expresses only a few latent proteins together with 12 pre-microRNAs. Previous microarray and proteomic studies predicted that multiple splice variants of the tumor suppressor protein tropomyosin 1 (TPM1) were targets of KSHV microRNAs. Here we show that at least two microRNAs of KSHV, miR-K2 and miR-K5, repress protein levels of specific isoforms of TPM1. We identified a functional miR-K5 binding site in the 3’ untranslated region (UTR) of one TPM1 isoform. Furthermore, the inhibition or loss of miR-K2 or miR-K5 restores expression of TPM1 in KSHV-infected cells. TPM1 protein levels were also repressed in KSHV-infected clinical samples compared to uninfected samples. Functionally, miR-K2 increases viability of unanchored human umbilical vein endothelial cells (HUVEC) by inhibiting anoikis (apoptosis after cell detachment), enhances tube formation of HUVECs, and enhances VEGFA expression. Taken together, KSHV miR-K2 and miR-K5 may facilitate KSHV pathogenesis.

## Introduction

In general adult populations, the prevalence of Kaposi’s sarcoma-associated herpesvirus is low in North and South America, Asia, and Northern Europe (5–10%), but more common in the Mediterranean region (20–30%) and common in sub-Saharan Africa (greater than 50%) [1]. In Northern Europe and the United States, prevalence is notably higher (20–40%) in populations with specific risk factors like immunodeficiency (e.g. HIV/AIDS) or homosexuality among men [2–4]. KSHV infection of B lymphocytes can lead to primary effusion lymphoma [5] and multicentric Castleman’s disease [6]. Kaposi’s sarcoma (KS) is a vascular tissue hyperplasia resulting from the infection of endothelial cells by Kaposi's sarcoma-associated herpesvirus (KSHV). Endothelial cells infected by KSHV undergo malignant transformation with high angiogenic activity [7, 8]. In most KS cells, KSHV is in latent phase and expresses only few viral proteins together with at least 18 mature KSHV microRNAs (miRNAs) arising from 12 pre-miRNAs [9]. To date, few targets of KSHV microRNAs (miR-Ks) have been investigated for associated functions [10–12]. During KS, a large rearrangement of the host cytoskeleton occurs [13], and two gene expression microarray assays have reported that the cytoskeletal protein tropomyosin 1 (TPM1) is down-regulated during KSHV infection of telomerase-immortalized microvascular endothelial (TIME) cells or lymphatic endothelial cells (LECs) [14, 15]. Additionally, cytoskeleton remodeling genes were enriched among predicted targets of EBV and KSHV miRNAs using PAR-CLIP [16] [12]. However, functions of TPM1 in KS remain unknown and no link has been established between miR-Ks and TPM1 expression in infected cells.

Mammalian tropomyosins are a vast family of actin binding proteins [17]. TPM proteins are divided in two groups according to their molecular weight: the low molecular weight (LMW) TPM (MW<30kDa) and the high molecular weight (HMW) TPM (MW>30KDa). All TPM isoforms (22 cloned isoforms in humans) are generated by alternative splicing of four distinct genes (TPM1 to 4) [18]. The TPM1 gene has two alternative promoters, two pairs of mutually exclusive exons and three polyadenylation sites. Consequently, the TPM1 gene potentially encodes 18 splice variants, 12 HMW isoforms and 6 LMW isoforms. In human, 11 TPM1 isoforms were identified so far (7 HMW and 4 LMW). However, expression of the HMW forms of TPM1 is abolished in many transformed cell lines and carcinoma, such as in breast carcinoma cell lines [19–21], in high-metastatic Lewis lung carcinoma [22] and in tongue squamous cell carcinoma [23], whereas expression of LMW-TPM isoforms are generally not affected during oncogenic transformation [24]. Nevertheless, forced expression of TPM1 in primary breast tumor cells restores anoikis [25] (apoptosis induced by loss of anchorage) and blocks malignant growth [26]. Consequently, TPM1 is commonly described as a tumor suppressor [24, 25, 27]. Interestingly, over-expression of the oncomir hsa-miR-21 in transformed cells could result in down-regulation of HMW-TPM1 [27, 28]. Moreover, it was proposed that the HMW forms of TPM1 and TPM2 translocate to the surface of endothelial cells that have been activated by growth factors, such as basic fibroblast growth factor (bFGF) or vascular endothelial cell growth factor (VEGF). At the cell surface, TPMs act as receptor for plasma ligands such as cleaved Kinigen (HKa) [29, 30], histidine-proline-rich glycoprotein (HPRG) [31, 32] and endostatin [33]. Neutralization of cell surface TPMs with an antibody directed against TPM1 and TPM2 blocks the anti-angiogenic activities of those ligands [34]. These reports suggest that TPMs may play a role in modulating angiogenesis.

Using gene expression profiling, we identified the HMW isoforms of TPM1 that are down-regulated during KSHV infection. We found that two miRNAs of KSHV, miR-K2 and miR-K5, repressed HMW-TPM1s expression in human umbilical vein endothelial cells (HUVECs). miR-K5 is able to target the 3’UTR of a HMW-TPM1 isoform while miR-K2 down-regulates HMW-TPM1s likely through targeting the coding sequence of TPM1 or an indirect mechanism. Furthermore, we confirmed that the expression of the HMW-TPM1s is consistently and robustly down-regulated during KSHV infection. Moreover, a locked nucleic acid (LNA) inhibitor of miR-K2 partially restored expression of HMW-TPM1s in endothelial cells infected by KSHV. We demonstrated that miR-K2 mimics decreased anoikis of HUVECs similar to the effects seen with siRNAs directed against TPM1. Finally, we observed an enhancement of endothelial cell tube formation by miR-K2 or siRNAs inhibiting TPM1. Together, these results give insights into how KSHV may promote KS pathogenesis by expressing miRNAs that reduce TPM1 protein levels in infected endothelial cells.

## Materials and Methods

### Cells and Reagents

Primary human umbilical vein endothelial cells (HUVECs) were used for no more than five passages in EGM-2 (Lonza). Human embryonic kidney (HEK)-293 cells and human cell line SLK [35] infected with recombinant r.KSHV.219 (SLKK) [36] were routinely cultured in Dulbecco's modified Eagle's medium at 4.5 g/L glucose supplemented with 10% fetal bovine serum and 1x ampicillin/streptomycin glutamine solution (Gibco). KSHV infected B cell line isolated from primary effusion lymphoma (BCBL-1) were routinely cultured in Roswell Park Memorial Institute medium at 4.5 g/L glucose supplemented with 15% fetal bovine serum and 1x ampicillin/streptomycin/glutamine solution (Gibco) and 55 μM ß-mercaptoethanol. The following mirVana KSHV miRNA mimics were obtained from Ambion: mirVana control (catalog number 4464060), mirVana miR-K2 mimic: 5’-AACUGUAGUCCGGGUCGAUCUG-3’, mirVana miR-K5 mimic: 5’-UAGGAUGCCUGGAACUUGCCGGU-3’. The following miRCURY locked nucleic acid (LNA) miRNA antisense inhibitors were obtained from Exiqon: LNA inhibitor control: 5’-GTGTAACACGTCTATACGCCCA-3’, LNA inhibitor of miR-K2: 5’-AGATCGACCCGGACTACAGT-3’, LNA inhibitor of miR-K5: 5’-CGGCAAGTTCCAGGCATCCT-3’. ON-TARGETplus small interfering RNA (siRNA) targeting exon-12 (5’-ACUUAGAAGACGAGCUGUAUU-3’) or exon-15 (5’-AUGACAAUCUGUAGGAUAAUU-3’) of tropomyosin1, and siRNA control ON-TARGETplus non-targeting “si-Neg” were obtained from ThermoScientific. Sponge vector against miR-K5 was generated as described by Gottwein et al. [37] Briefly, 9 imperfect miR-K2 binding sites were introduced in the 3’UTR of the eGFP reporter of a pLenti/CMV/eGFP vector (Addgene) by using the following primers: 5’-CTAGCAGATCGACCCCCTCTACAGTTGTTTTGCAGATCGACCCCCTCTACAGTTGTTTTGCAGATCGACCCCCTCTACAGTTTCTAGATTTGAATTC-3’ and 5’-AATTGAATTCAAATCTAGAAACTGTAGAGGGGGTCGATCTGCAAAACAACTGTAGAGGGGGT CGATCTGCAAAACAACTGTAGAGGGGGT CGATCTG-3’. The sequences of the primers use to detect TPM1 isoforms by qPCR are:

Primer4 (forward primer binding exon IV) 5’-CCACGAGAGGAAGCTGA-3’; Primer5 (reverse primer binding exon V) 5’-CCAACTCTTCCTCAACCA-3’; Primer8 (forward primer binding exon VIII) 5'-GCATTAATGGCTGCAGAG-3'; Primer15 (reverse primer binding exon XV) 5'-TTTCTTCTTTGGCATGAGC-3'. Taqman probes from Applied Biosystems were used to detect miR-K2 (catalogue number 197192_mat) and miR-K5 (catalogue number 464502_mat).

### Transfection

HUVECs were transfected with 20 nM of siRNA or 10 nM of miRNA or 50 nM of LNA using Dharmafect1 (Dharmacon). Approximately 24 hours before transfection, 2x10^5^ cells/well were seeded in 6-well plates. Thirty minutes before transfection, the cell medium was replaced with 2.4 ml of fresh medium. The transfection mix was prepared by adding 1.5 μl of Dharmafect1 to 300 μl of Opti-MEM (Gibco) and incubated at room temperature (RT) for 5 min. Then, Dharmafect solution was mixed with 300 μl of Opti-MEM containing 30 pmoles of miR-K or 60 pmoles of siRNA. Six hundred μl of transfection mixes were incubated for 20 min at RT before addition to the cells. For plasmid and miRNA co-transfection, HUVECs in 6-well plates were transfected using Lipofectamine LTX with PLUS reagent and 2.1 μg of plasmid and 30 pmoles of miRNAs. Fresh media with 400 μg/ml G418 was added 24 hr. after transfection (hpt) and cells were harvested 3 days after transfection.

### Quantitative Western Blot

Cell pellets were incubated on ice for 20 min in radioimmunoprecipitation assay (RIPA) lysis buffer (Sigma) supplemented with protease and phosphatase inhibitor cocktail (Thermo Scientific). Cell debris was removed by 10,000 g centrifugation at 4°C for 30 min. Protein concentration of the supernatant was evaluated using Quick Start Bradford Dye Reagent 1X (Bio-Rad). 28 μg of protein was separated using a 1-mm NuPAGE 4–12% Bis-Tris Gel (Invitrogen) and transferred onto a nitrocellulose membrane using an iBlot (Invitrogen). The membrane was incubated for 30 min at RT in Odyssey blocking buffer (Li-Cor). Primary antibodies were incubated with the membrane overnight at 4°C, whereas secondary antibodies were incubated at RT for 2 hours. The Odyssey scanner (Li-Cor) was used for the detection and quantification of proteins. Primary antibodies used were: rabbit anti-TPM1 (catalog number 3910; Cell Signaling), rabbit anti-hnRNPH1 (Novus) and mouse anti-actin (catalog number A5316; Sigma) and mouse anti-GAPDH (catalog number sc-69778; Santa Cruz). Secondary antibodies conjugated to infrared fluorescing dyes were obtained from Li-Cor: goat anti-rabbit IR800CW (catalog number 926–32211) and goat anti-mouse IR680RD (catalog number 926–68070). Results are presented as TPM1 expression normalized to actin, relative to mock-infected or negative-control mi/siRNA-transfected cells.

### Luciferase Assays

Each 3’UTR of TPM1 isoforms were cloned into the 3'UTR of the renilla luciferase reporter whose expression is controlled by the herpes simplex virus TK promoter. This work was performed by the Protein Expression Laboratory (SAIC, Frederick, MD). As an internal control, these vectors express the firefly luciferase driven by the simian virus 40 promoter. A luciferase reporter without UTR insertion served as a control for any non-specific luciferase expression in response to the KSHV miRNAs. To mutate the luciferase reporter carrying exon-13, the QuikChange II kit (Stratagene) was used according to the manufacturer’s instructions with the following primers: 5'-ACCCTGGTTCTCTCTCTTAGC AAAATGCCTTAGAGCCAGGC-3' and 5'-GCCTGGCTCTAAGGCATTTTGCTAAGAGA GAGAACCAGGGT-3'. Luciferase assays were performed as described in Abend et al. [38]. Briefly, in a 96-well plate, 3x10^4^ HEK-293 cells were reverse transfected using 75 ng of luciferase reporter vector, 13.3 nM of mimic miR-K, and 0.5 μl of Lipofectamine 2000 (Invitrogen) in a final volume of 150 μl. Similarly, 3x10^4^ SLK-K cells were reverse transfected using 25 ng of luciferase reporter vector and 0.5 μl of Lipofectamine 2000 (Invitrogen) in a final volume of 150 μl. Also, 2x10^6^ BCBL-1 cells were electroporated with 25ng of luciferase reporter in 400 μl of RPMI in a Gene Pulser Xcell (Bio-Rad) set at 220V, 975 μF, and exponential wave function. After the pulse, BCBL-1 cells were placed in 4 ml of complete medium in a 6-well plate. After transfection at the indicated time, cells were lysed with passive lysis buffer (Promega). Assays were performed using the Dual Luciferase reporter system (Promega) Luciferase activity from approximately 12,000 HEK-293/SLK-K and 125,000 BCBL-1 cells were measured. Each transfection was assayed at least three times and transfections of HEK-293/SLK-K cells were executed in triplicate.

### KSHV Production and *de novo* Infection

To produce KSHV, BCBL-1 cells were treated with 300 μM of valproic acid for 6 days. The cell supernatant was filtered through a 45-μm filter flask (SFCA membrane), and centrifuged at 25000g for 2 hours, at 4°C. The virus-containing pellet was re-suspended in EGM-2 containing 8 μg/ml Sequabrene (Sigma) and stored at -80°C. HUVECs grown to 60 to 70% confluence were infected with KSHV diluted in EGM-2 containing 8 μg/ml sequabrene for 6 hours at 37°C. Cells were washed twice with PBS, and medium was changed every day throughout the course of infection. The viruses used to infect HUVECs gave approximately 80% of primary infection efficiency as monitored by staining of the viral latency-associated nuclear antigen (LANA) 2 days post-infection (dpi, data not shown). SLK cells latently infected with BAC16 mutants of KSHV were provided by Rolf Renne and were maintained in DMEM with 10% FBS, 250 μg/mL G418, 1.2 mg/ml HygB, 1 μg/mL Puromycin.

### Ethics Statement

Lymph node biopsies were obtained from patients with lymphadenopathic KS and with confirmed history of HIV and KSHV co-infection. All patients were enrolled in an Institutional Review Board approved protocol at the National Cancer Institute (NCTNCT00006518, PI: Robert Yarchoan). All patients gave written informed consent. The presence of KSHV in the different samples was confirmed by immunohistochemistry staining of LANA. Clinical samples were homogenized and lysed in RIPA lysis buffer. Soluble protein was analyzed by Western blotting and TPM1 levels were normalized to GAPDH levels.

### Anoikis Assays

At 24 hpt, HUVECs were harvested and counted. Cells (5x10^4^ cells/well) were seeded in 1 ml of medium on a poly hydroxyethyl methacrylic acid (polyHEMA) coated 24-well plate (final density ≈1.3 mg of polyHEMA per cm^2^) and in a 24-well plate control. After 24 and 48 hours (48 and 72 hpt), cells were harvested and concentrated by centrifugation. Cell pellets were re-suspended in 100 μl of fresh medium and stained with calcein AM (Invitrogen) or WST-1 (Roche), according to the manufacturer’s instructions. After an hour, relative fluorescence units (RFU) for calcein AM (excitation/emission: 495/515 nm) or absorbance (A450-A650 nm) for WST-1 were read with a Modulus microplate reader (Promega). Results were presented as RFU or absorbance measurements in the polyHEMA-coated wells normalized to the control well, relative to mock-infected or negative-control mi/siRNA-transfected well.

### Tube Formation Assay

At 36 hpt, 20,000 HUVECs in EBM-2 (Lonza) complemented with 10% of EGM-2 were seeded in a 4-mm-diameter well (Ibidi μ-plate catalog number 81501) coated with Geltrex (Invitrogen). After 12 hours (48 hpt), the cells were stained with calcein AM (3 μg/ml) for an hour. The cells were washed twice with PBS and maintained in PBS during imaging. Tile images, covering the entire sample well, were acquired using the MosaiX module of Zeiss AxioVision 4.8 software controlling a Zeiss AxioObserver Z1 epifluorescence microscope (Carl Zeiss Microscopy) equipped with a 10x Plan-Neofluar Ph1 (N.A. 0.30) objective lens and a Photometrics CoolSnapES CCD camera (Photometrics). The tile images were stitched together and analyzed using the tube formation module in MetaMorph 7.7 software (Molecular Devices).

### Statistical Analysis

Data presented in this article are considered statistically relevant if the p-value of a two-tailed unpaired student t-test was lower than 0.05. Experiments used at least three biological replicates and experimental conditions were compared to the appropriate negative control. For Western blot analysis, the proteins of interest were first normalized to the internal loading standard (actin, GAPDH), then this ratio was normalized to the same ratio from the negative control experiment, (TPM/actin)_test_/(TPM/actin)_control_. This calculation yielded the relative change in gene expression, relative to the negative control expression. For the microarray analysis, linear LOWESS normalization was used. Default Agilent normalization was employed within Genespring GX.

## Results

### KSHV represses HMW-TPM1s

**Fig 1:**
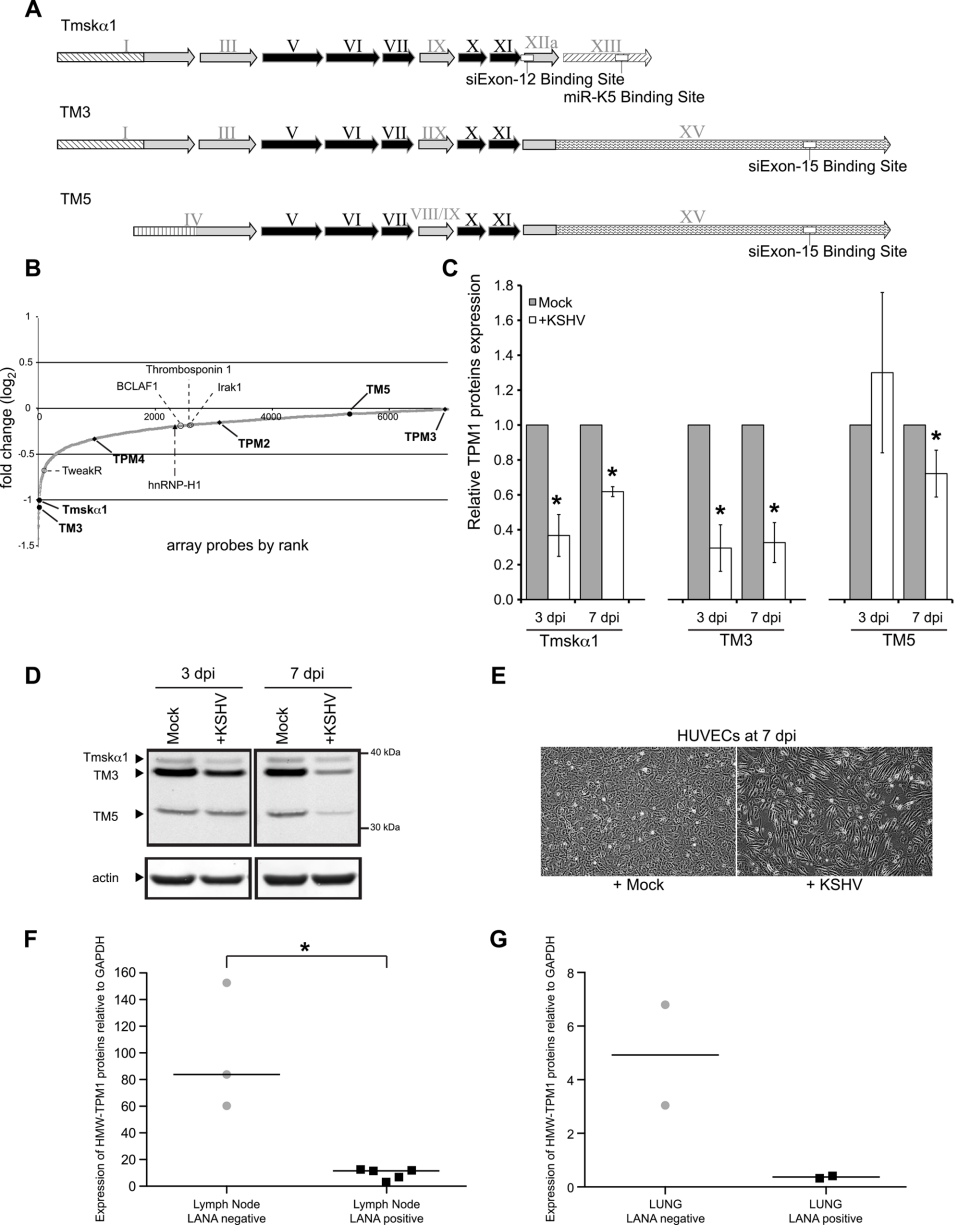
TPM1 protein expression is repressed in infected cells. **(A)** Schematic overview of the three TPM1 isoforms detected in HUVECs. Exons are indicated by Roman numerals. Exons common to all TPM1 isoforms are shaded in black whereas alternatively spliced exons are shaded in grey. The different untranslated regions (UTR) sequences are designated by different striped patterns on a white background. White boxes indicate binding region of miR-K5 or siRNAs. HMW, high-molecular weight; LMW, low-molecular weight. **(B)** Host mRNA expression screen data in latently infected HUVECs (48 hpi). Additionally, the fold expression change of several known targets of KSHV miRNAs and other members of tropomyosin family are indicated. **(C)** Average TPM1 protein expression levels in KSHV *de novo* infected HUVEC normalized to actin and relative to levels of mock infected cells based on six independent experiments (representative blot in panel D). Statistically significant data (p<0.05) are indicated with an asterisk. **(D)** Immunoblotting of TPM1 with total protein extracts in HUVEC and *de novo* KSHV infected HUVEC at 3 and 7 dpi. Two HMW-forms (Tmskα1, TM3) and one LMW-form (TM5) of TPM1 were detected using Western blotting. Tmskα1 and TM3 are repressed a few days after infection, whereas TM5 expression is altered at a later time point. **(E)** At 7 dpi, spindle cells were visible in KSHV infected population. **(F)** Quantification of HMW-TPM1 in LANA negative or positive lymph node biopsies from patients with lymphadenopathic KS, or patients without KS. **(G)** Quantification of HMW-TPM1 in LANA negative or positive lung biopsies from patients with or without KS.

The TPM1 gene encodes multiple splice variants (Fig 1A). Thus far, two high molecular weight (HMW) variants were identified in HUVECs, Tmskα1 and TM3 [39, 40]. To identify host targets of KSHV miRNAs, we used microarray data from HUVECs latently infected with KSHV [41]. It appeared that the HMW-TPM1 isoforms were consistently down-regulated during KSHV infection (Fig 1B). In our recent proteomics analysis to identify KSHV miRNA targets in HUVECs [42], HMW-TPM1 protein levels were repressed in the presence of KSHV miRNAs (log_2_ = -0.26, 125^th^ most repressed of 1271 proteins). In protein extracts of HUVECs, we detected a low molecular weight (LMW) variant of TPM1 (Fig 1C). RNA interference experiments coupled with the amplification of a specific region of TPM1 mRNA allowed us to identify the LMW-TPM1 as either TM5a or TM5b (data not shown, referred as TM5 here) [43]. To identify which TPM1 variants were repressed in the context of KSHV infection, we infected HUVECs with KSHV and harvested the cells at 3 and 7 days post-infection (dpi). Thenceforth, we detected a severe reduction of HMW-TPM1 protein expression compared with mock-infected cells (Fig 1C and 1D). Down-regulation of HMW-TPM1 proteins persisted during the latent phase of infection. Indeed, at 7 dpi, when spindle cells were easily identified (Fig 1E), the expression levels of HMW-TPM1 isoforms were still significantly lower than in mock-infected cells. Finally, we analyzed TPM1 expression in fresh biopsies of KS patients. In the five LANA-positive lymph nodes from KS patients that we examined, HMW-TPM1 proteins were weakly expressed compared with the LANA-negative lymph nodes (Fig 1F). HMW-TPM1 proteins are also expressed at lower levels in limited fresh lung biopsies infected by KSHV compare with the LANA-negative lung samples (Fig 1G). Together, these data show that HMW-TPM1 isoforms are down-regulated during KSHV infection.

### miR-K5 targets the 3’UTR of Tmskα1

**Fig 2:**
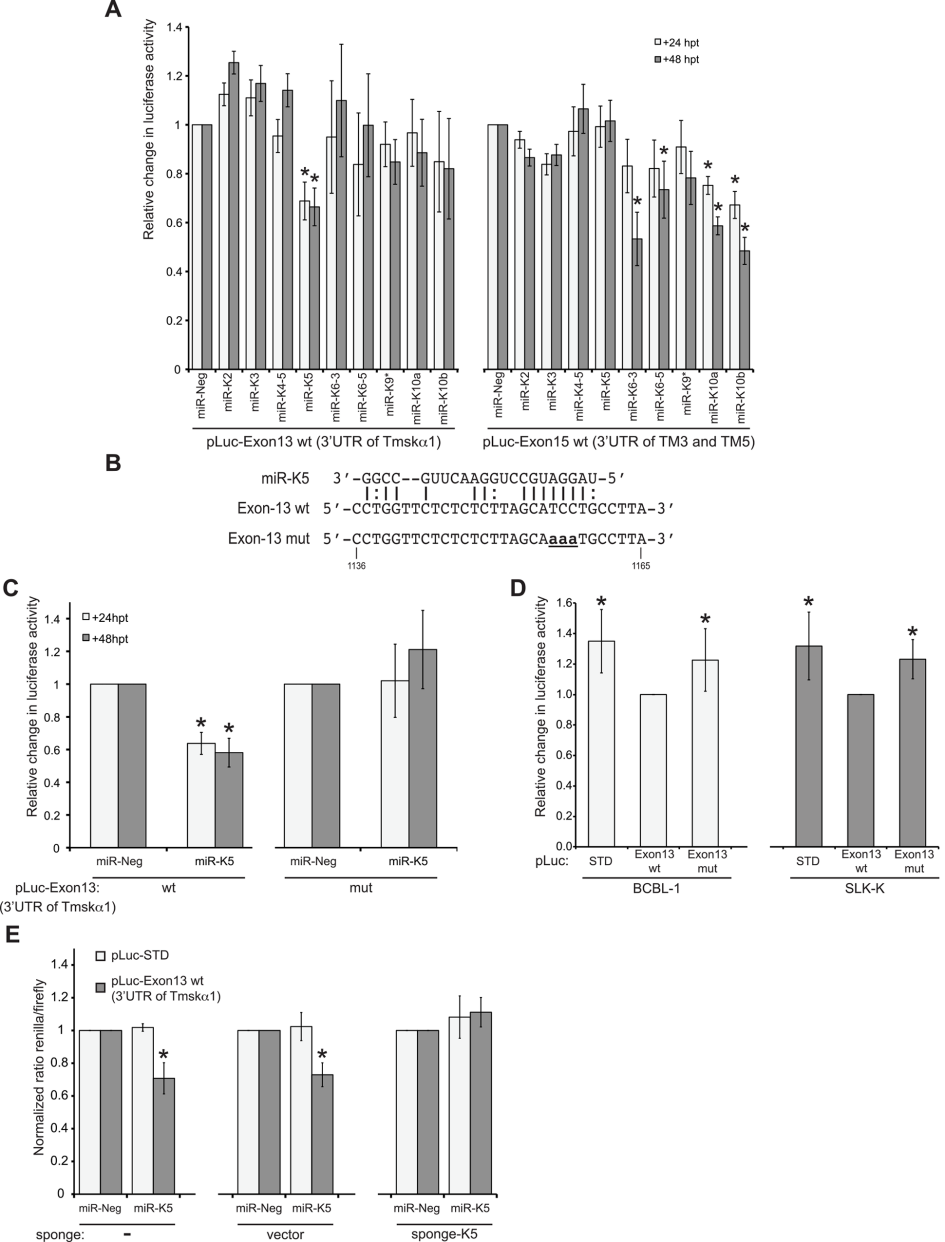
miR-K5 targets the 3'UTR of Tmskα1. **(A)** Exon-13 and -15, which encode the different 3'UTRs of TPM1 isoforms were cloned in a luciferase reporter and transfected in HEK-293 cells in the presence of individual miRNAs. Luciferase activity was measured at 24 and 48 hpt. Results are presented as the change in normalized RFU relative to negative-control miRNA (miR-Neg). **(B)** miRanda identified a putative binding site for miR-K5 in the 3'UTR of Tmskα1 encoded by exon-13. The luciferase reporter carrying exon-13 was mutated as indicated (**aaa**). **(C)** Luciferase assays were performed with miR-Neg or miR-K5 cotransfected with the luciferase reporter carrying the wild-type (pLuc-Exon13 wt) or the mutated exon-13 (pLuc-Exon13 mut). **(D)** The parental luciferase reporter (pLuc-STD) and the luciferase reporter carrying the wild-type or mutant exon-13 were transfected in cell lines constitutively expressing KSHV miRNAs. At 48 hpt, luciferase activity was measured and expressed relatively to the luciferase activity of pLuc-Exon13 wt. **(E)** At 24 hpt, luciferase activity of the reporter carrying the wild-type exon-13 was compared with the luciferase activity of the backbone reporter vector (pLuc-STD) co-transfected with miR-Neg or miR-K2 in the presence of a sponge vector without a binding site for miR-K5 (vector) or with nine imperfect binding sites for miR-K5 (sponge-K5). All results were based on at least three independent experiments. Statistically significant repression of luciferase activity is indicated with an asterisk.

Since we observed reduced HMW-TPM1 protein expression during KSHV infection, we investigated whether down-regulation of HMW-TPM1 could be caused by KSHV miRNA expression. Usually miRNAs bind in the 3’UTR of target mRNAs. Therefore, we cloned the 3’UTRs of TPM1 isoforms downstream of a luciferase reporter and performed a luciferase assay in the presence of KSHV miRNA mimics in HEK-293 cells (Fig 2A). Exon-13 of TPM1 gene encodes the 3’UTR of Tmskα1, while exon-15 encodes the 3’UTR of TM3 and TM5. Only miR-K5 was able to repress the expression of the luciferase reporter containing the exon-13 of TPM1 gene (pLuc-Exon13 wt) at 24 and 48 hpt. Sequence analysis of TPM1 3’UTRs with miRanda [44] revealed a putative binding site for miR-K5 in exon-13. We mutated the luciferase reporter carrying exon-13 by changing three nucleotides in the region recognized by the seed of miR-K5 (pLuc-Exon13mut, Fig 2B). Again, luciferase activity of the reporter carrying the wild type exon-13 was reduced in the presence of miR-K5 mimics (Fig 2C). In contrast, miR-K5 mimics had no effect on luciferase activity of the mutated reporter. In addition to miRNA mimics, we used cell lines constitutively expressing KSHV miRNAs, such as SLK-K [36] or BCBL-1 [45], to perform luciferase assays measuring endogenous miRNA activity. Inhibition of the luciferase activity of the reporter carrying the wild type exon-13 is rescued when the binding site of miR-K5 is mutated (Fig 2D). Finally, a “sponge” vector carrying nine binding sites for miR-K5 inhibited repression of Exon-13 by miR-K5 (Fig 2E). Rescue of luciferase activity by mutating the miR-K5 binding site on the luciferase reporter or by quenching miR-K5 mimics with a “sponge” vector demonstrated the specificity of miR-K5 for the 3’UTR of Tmskα1.

### miR-K2 and miR-K5 decrease the protein level of HMW-TPM1 isoforms in HUVECs

**Fig 3:**
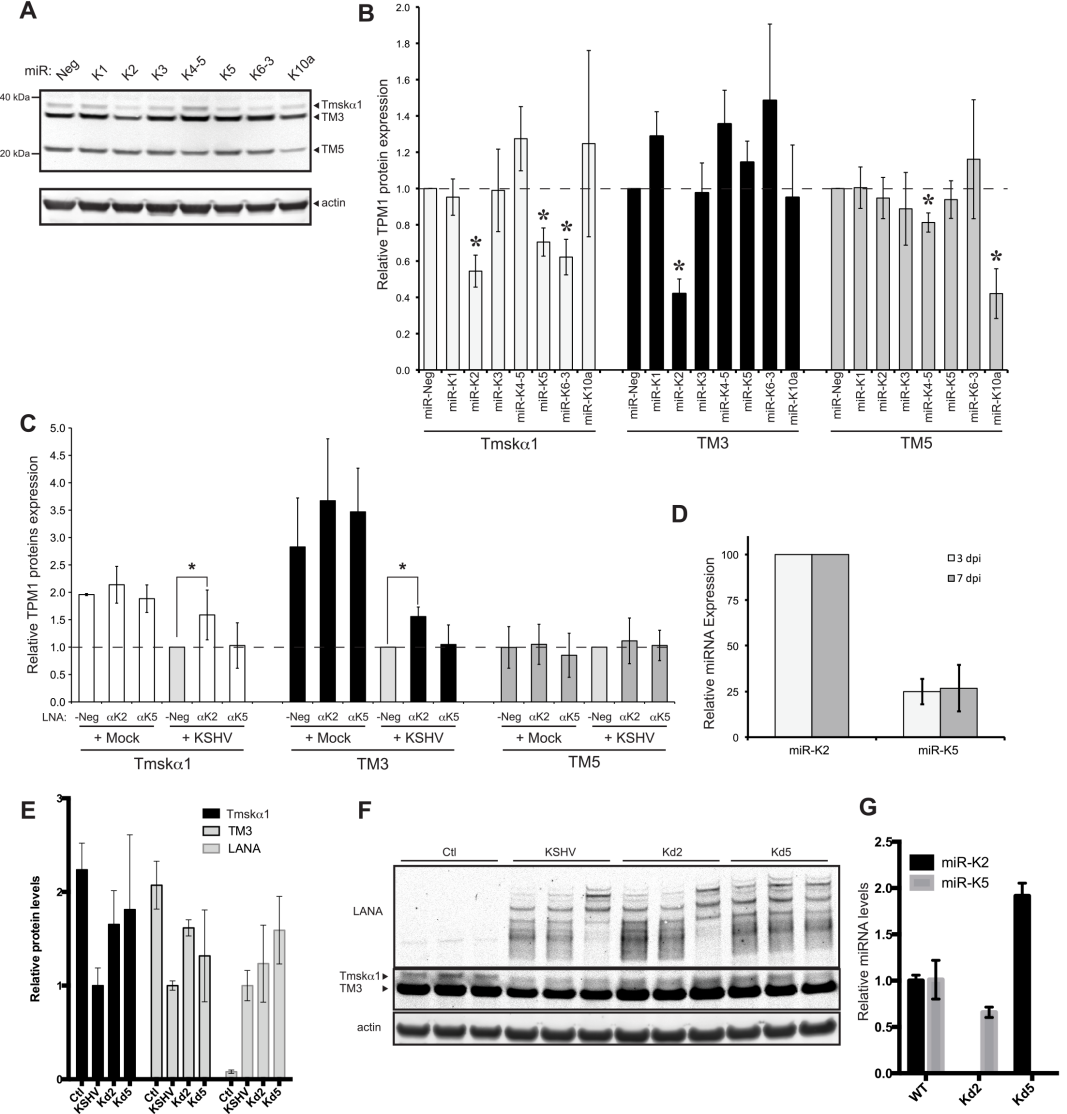
miR-K2 and miR-K5 down-regulate the expression of HMW-TPM1. HUVECs were transfected with individual KSHV miRNA. After 48 hours, expression of TPM1 and actin was analyzed by quantitative Western blot. **(A)** Representative images for TPM1 and actin expression are shown. **(B)** The average change in TPM1 protein expression levels was normalized to actin and to TPM1 levels in cells transfected with miR-Neg (based on four independent experiments). **(C)** HUVECs were transfected with a LNA control (LNA-Neg), inhibitor of miR-K2 (LNAαK2), or inhibitor of miR-K5 (LNAαK5). At 24 hpt, the cells were infected with KSHV for 48 more hours. After 72 hpt (2 dpi), total cell lysates were harvested and the expression of TPM1 was analyzed by quantitative immunoblot. Results are presented as the average change in TPM1 protein expression levels normalized to actin and relative to the levels of LNA-Neg in *de novo* infected cells. Average and SD values were calculated from four independent experiments. **(D)** Relative KSHV miRNA expression level determined by Taqman qPCR in *de novo* infected HUVEC. (E) Relative protein level changes from cells infected with wild-type KSHV or mutant version lacking miR-K2 (Kd2) or mirR-K5 (Kd5). (F) Immunoblot for data in (E). (G) Relative miRNA level changes from cells infected with mutant viruses used in (E) and (F).

We identified KSHV miRNAs that were able to repress luciferase reporters carrying the 3’UTRs of TPM1. To confirm that TPM1 proteins are targets of KSHV miRNAs in HUVECs, we performed a series of transfection experiments using individual KSHV miRNA mimics and monitored protein expression levels of TPM1 at 48 hpt by quantitative western blot (Fig 3A). As expected, miR-K5 mimics significantly down-regulated Tmskα1 protein expression. Remarkably, of all KSHV miRNA mimics tested, the miR-K2 mimic was the only one capable of down-regulating both HMW-TPM1 isoforms expressed in endothelial cells (Fig 3B). We showed that miR-K5 targeted the 3’UTR of Tmskα1 and miR-K5 mimics repressed the protein level of Tmskα1 in HUVEC. Whereas miR-K2 does not target the 3’UTRs of HMW-TPM1, miR-K2 mimics decreased the protein level of HMW-TPM1s expressed in HUVECs. It was reported that transfection of miRNA mimics resulted in similar levels of RISC-associated miRNA mimics as endogenous miRNAs [46]. Hence, down-regulation of TPM1 by miR-K2 and miR-K5 mimics should reproduce the behavior of KSHV miRNAs during KSHV infection. Consequently, we next investigated whether the down-regulation of HMW-TPM1 during KSHV infection of HUVEC was due to miR-K2 and miR-K5. Therefore, we evaluated the impact of the inhibition of miR-K2 and miR-K5 on TPM1 protein levels. HUVECs previously transfected with locked nucleic acid (LNA) miRNA antisense inhibitors for miR-K2 or miR-K5 or a control LNA were later infected with KSHV. In infected cells transfected with the LNA control, we detected a down-regulation of Tmskα1 and TM3, whereas the TM5 level remained unchanged compared with mock infected cells. However, the expression of Tmskα1 and TM3 was significantly higher in infected cells in the presence of LNA inhibitors of miR-K2, compared with the LNA negative control inhibitor (Fig 3C). This showed that the repression of Tmskα1 and TM3 induced by KSHV is inhibited by the LNA directed against miR-K2. These findings are consistent with the down-regulation of Tmskα1 and TM3 observed in HUVECs transfected with miR-K2 mimics. Surprisingly, although we demonstrated a direct binding of miR-K5 to the 3’UTR of Tmskα1 and we showed that miR-K5 down-regulated Tmskα1 in HUVECs, the LNA inhibitor of miR-K5 failed to rescue expression of Tmskα1 during KSHV infection. This could be due to incomplete inhibition of miR-K5 by LNAs or to the low expression of miR-K5 relative to miR-K2 after *de novo* infection (Fig 3D).

We also used a complementary approach utilizing cells infected with wild type KSHV or mutant forms lacking miR-K2 or miR-K5. In these assays, de-repression was observed for Tmskα1 when miR-K5 was deleted (Fig 3E–3G). Additionally, Tmskα1 and TM3 levels increased when miR-K2 is deleted. The various validation assays in Table 1 contained some inconsistencies across the different assays. These may have been caused by the different measurements, such as steady-state endogenous protein levels versus ectopic luciferase reporter genes. Other potential explanations included miRNAs targeting outside of the cloned 3’UTR initially tested and/or indirect mechanisms of repression by miRNAs. However, multiple assays support the conclusion that expression of HMW-TPM1 isoforms is repressed by miR-K2 and miR-K5.

**Table 1:** TPM1 isoforms expressed in HUVECs.

	MW	Expression during infection	Expression rescued during infection by	3’UTR encoded by	3’UTR luciferaser reporter repressed by	3’UTR contains binding site for	Protein level repressed by	Effective siRNA
**Tmsk**α**1**	HMW	**- -**	LNAαK2	Exon-13	miR-K5	miR-K5	miR-K2	si-Exon12
							miR-K5	
							miR-K6-3	
**TM3**	HMW	-—-	LNAαK2	Exon-15	miR-K6-3	N/A	miR-K2	si-Exon12
					miR-K6-5			
					miR-K10a			si-Exon15
					miR-K10b			
**TM5**	LMW	**-**	N/A	Exon-15	miR-K6-3	N/A	miR-K4-5	si-Exon15
					miR-K6-5			
					miR-K10a		miR-K10a	
					miR-K10b			

Summary of the different features of TPM1 isoforms expressed in HUVEC. MW: molecular weight, HMW: High molecular weight, LMW: Low molecular weight, “-“: repression.

### miR-K2 inhibits expression of HMW-TPM1s outside of the 3’UTR

**Fig 4:**
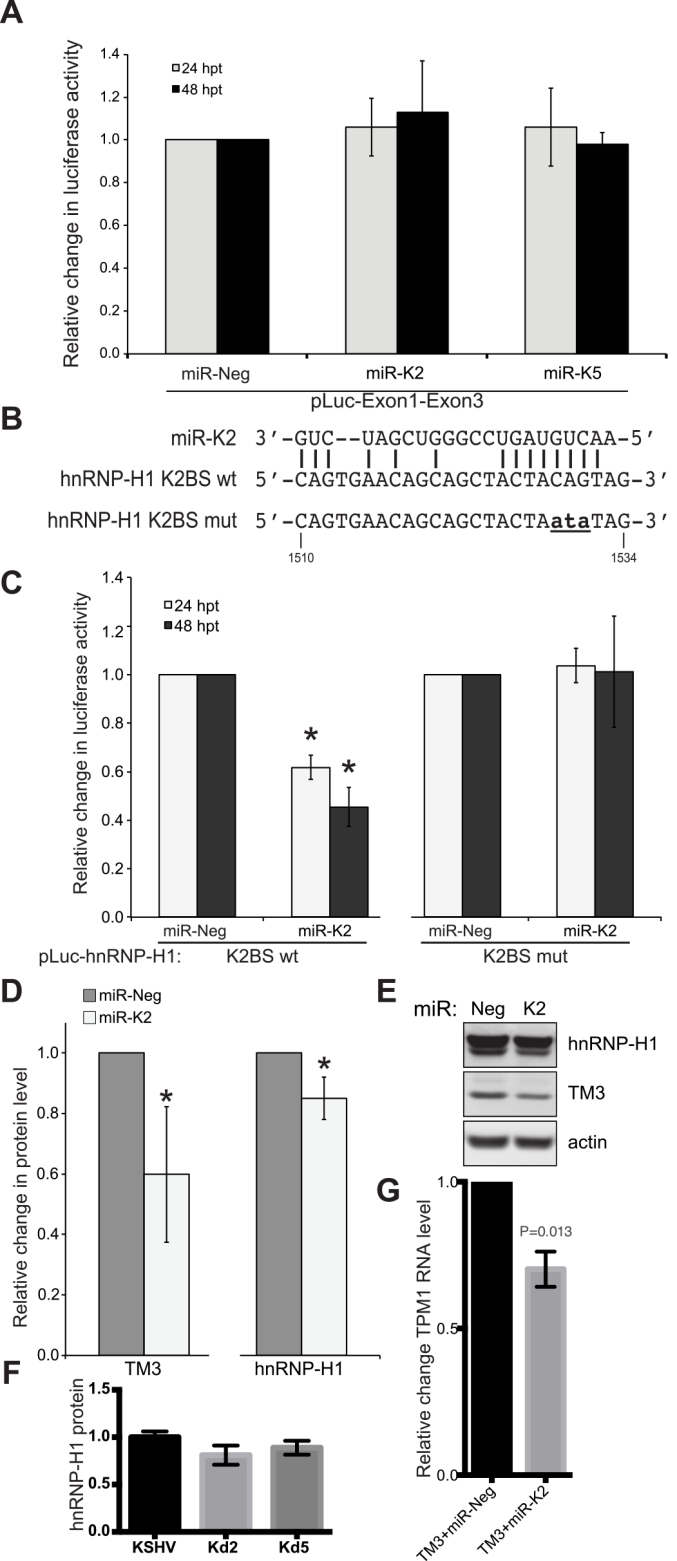
miR-K2 indirectly regulates expression levels of HMW-TPM1s. **(A)** Luciferase reporter carrying the exon-1 and exon-3 of TPM1 gene (pLuc-Exon1-Exon3) was transfected in HEK-293 cells in the presence of miR-K2 mimics, miR-K5 mimics or a mimic control (miR-Neg). Luciferase activity was measured at 24 and 48 hpt. Results are presented as the change in normalized RFU relative to negative-control miRNA (miR-Neg). Average and SD were calculated from three independent experiments. **(B)** The putative binding site for miR-K2 in the 3'UTR of hnRNP-H1 was introduced downstream of a luciferase reporter (pLuc-hnRNP-H1 K2BS wt). The same oligonucleotide with three base pairs substitutions (**ata**) in the seed-matching region of miR-K2 was also introduced in a luciferase reporter (pLuc-hnRNP-H1 K2BS mut). **(C)** Co-transfection in HEK-293 of miR-K2 mimics with the luciferase reporter carrying the wild type or mutated sequence of the putative miR-K2 binding site of hnRNP-H1 3’UTR. Luciferase activity was measured 24 and 48 hpt. Results are presented as the change in normalized RFU relative to negative-control miRNA (miR-Neg). Average and SD values were calculated from four independent experiments. (D) HUVECs were transfected with a control (miR-Neg) or miR-K2 mimics and protein levels were measured by quantitative immunoblotting in six experiments. Statistically significant repression (p < 0.05) is indicated with an asterisk. (E) Representative image for hnRNP-H1, TM3 and actin expression is shown. (F) Protein levels in immunoblots (n = 3) from cells infected with wild-type KSHV, mutant version lacking miR-K2 (Kd2) or mirR-K5 (Kd5). (G) TPM1 RNA levels after co-transfection with TM3 cDNA plasmid and miRNA control (Neg) or miR-K2.

In HUVECs, miR-K2 repressed protein expression of Tmskα1 and TM3, but did not affect TM5 (Fig 3). However, the luciferase assays did not detect repression by miR-K2 of the luciferase reporters containing the 3’UTR of Tmskα1 and TM3 (Fig 2A). Consequently, miR-K2 was expected to bind to a region other than the 3’UTR of Tmskα1 and TM3. We hypothesized that miR-K2 target sequences could be exon-1 or exon-3 since these are the only exons shared between Tmskα1 and TM3, but absent from TM5 mRNA transcripts (Fig 1A). To test this hypothesis we performed a luciferase assay with a reporter carrying the sequence of exon-1 and -3 in the presence of miR-K2 or miR-K5 mimics. We did not detect significant change in the activity of the luciferase reporter containing the exon-1 and -3 compared with the reporter control (Fig 4A). Consequently, we hypothesized that miR-K2 inhibits expression of HMW-TPM1s by an indirect mechanism. The indirect effect of miR-K2 on HMW-TPM1s could be explained by the regulation of splicing factors. Indeed, splicing of HMW-TPM1s requires specific factors because the splicing of exon-2 and -3 of TPM1 gene is mutually exclusive. Previous data showed that enhancing the splicing of exon-2 diminishes the amount of Tmskα1 and TM3 since their transcripts possess exon-3 [47]. Several splicing factors were described as specific regulators of the splicing of exon-2 and -3 [17, 48], including hnRNP-H1. Knock-down of hnRNP-H1 expression led to a decrease in exon-3 transcript [47], consistent with the TPM1 isoform expression pattern induced by miR-K2. Furthermore, hnRNP-H1 was down-regulated during KSHV latent infection of HUVECs according to mRNA expression profiling arrays (Fig 1B). Interestingly, previous PAR-CLIP experiments identified a miR-K2 binding site in the 3’UTR of hnRNP-H1 [16]. To investigate a possible targeting of hnRNP-H1 3’UTR by miR-K2, we cloned the putative wild-type (K2BS wt) or mutated (K2BS mut) miR-K2 binding sequences from the hnRNP-H1 3’UTR into a luciferase reporter (Fig 4B). The miR-K2 mimics strongly reduced the luciferase activity of the reporter carrying the wild type binding site, but the reporter with the mutated site was not repressed by miR-K2 (Fig 4C). In addition, hnRNP-H1 protein levels were modestly repressed in primary endothelial cells transfected with miR-K2 mimics (Fig 4D and 4E). However, when KSHV-infected cells lack miR-K2, hnRNP-H1 protein levels did not increase (Fig 4F), suggesting that miR-K2 does not strongly down-regulate the expression level of hnRNP-H1 during infection.

Additionally, we co-transfected a cDNA plasmid encoding TM3 and miR-K2 into primary endothelial cells. We observed an average 43-fold induction of the TM3 transcript, but this activation was repressed an average 30% (Fig 4G) when co-transfected with miR-K2 (relative to a negative control miRNA). The level of repression is likely underrepresented since the TM3 overexpression was so pronounced. Since the transfected TM3 plasmid lacks the 3’UTR of the endogenous messenger RNA, this data along with the luciferase data in Fig 2 suggests that miR-K2 may repress TM3 expression by targeting an unidentified sequence in the protein coding region of TM3 transcript.

### KSHV infection and miR-K2 inhibit anoikis of HUVECs

**Fig 5:**
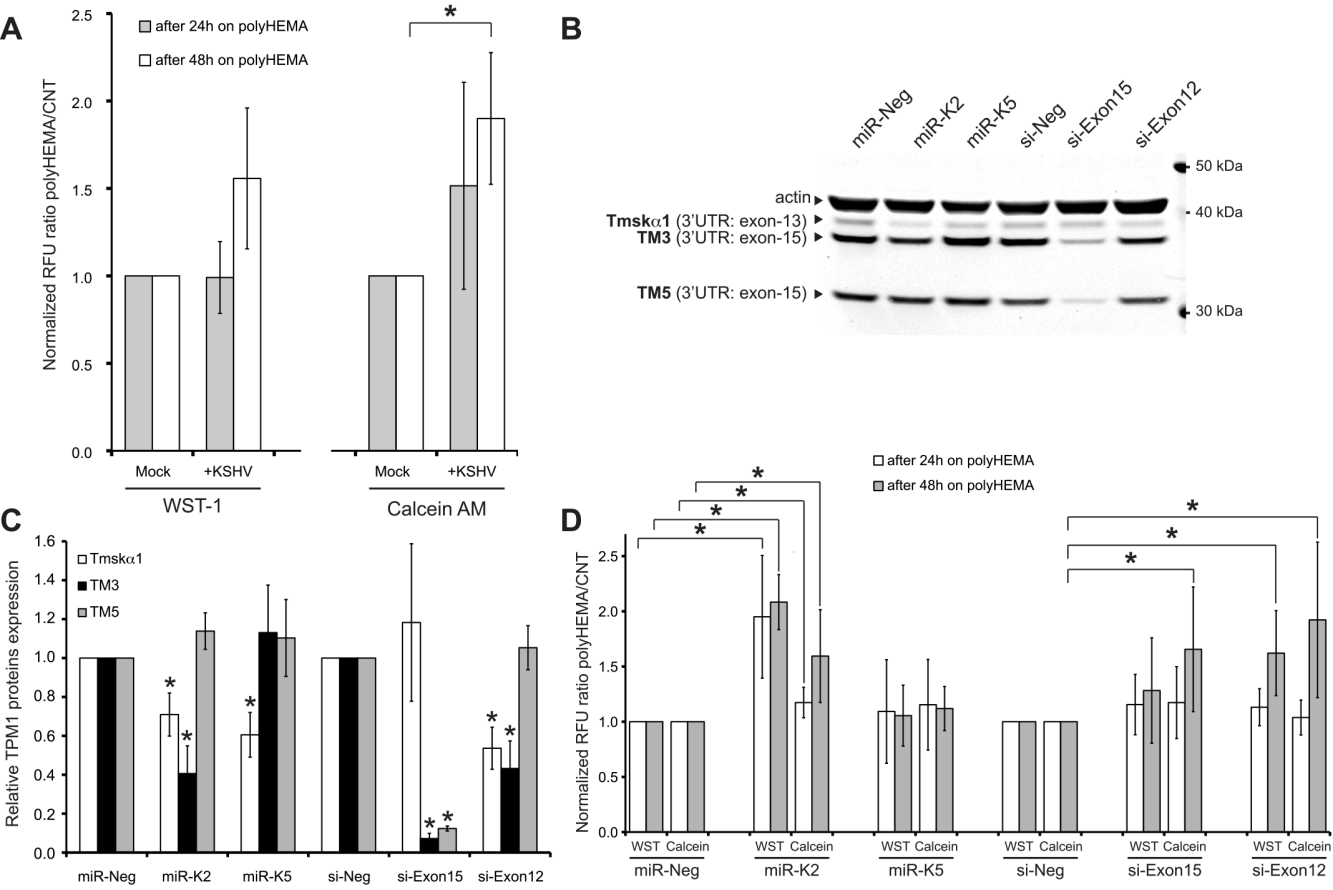
KSHV and miR-K2 decrease anoikis of endothelial cells. **(A)** At 7 dpi, HUVECs were transferred to a polyHEMA coated or a control 12-well plate. After 24 and 48 hours in the 12-well plate, cell viability was measured using WST-1 and calcein AM. Results are presented as the ratio of the viability signals polyHEMA/control (CNT) relative to mock-infected cells. Average and SD values were calculated from five independent experiments, with statistically significant data (p<0.05) indicated by an asterisk. **(B)** Representative immunodetection of TPM1 in total protein extract of HUVECs transfected 48 hours with the indicated RNA. **(C)** Quantification of TPM1 proteins at 48 hpt presented as the average change in TPM1 protein expression level normalized to actin and relative to levels of miR-Neg or si-Neg transfected cells and based on four independent experiments. **(D)** HUVECs transfected for 24 hours with the indicated RNA were transferred to a polyHEMA coated or a control 12-well plate. After 24 and 48 hours in the 12-well plate, cell viability was measured with the same process as in 5A.

TPM1 is not only involved in stabilization of actin stress fibers. In the absence of anchorage, adherent cells such as endothelial cells undergo a specific apoptotic program called anoikis [49, 50]. Surprisingly, miR-K2 and miR-K5 did not disturb the actin network [51], and have a modest impact on cells migration (data not shown). Because loss of TPM1 expression in breast cancer cells abolishes anoikis [25], and the forced expression of the KSHV protein vFLIP inhibits anoikis [52], we hypothesized that KSHV-infected cells are partially resistant to this particular type of cell death. Therefore, we seeded KSHV-infected HUVECs in wells coated with poly-2-hydroxyethyl methacrylate (polyHEMA), a hydrophobic polymer prohibiting attachment of the cells. Viability of cells was measured using WST-1 or calcein-AM (Fig 5A). Calcein-AM showed that the viability of HUVECs infected by KSHV was 90% higher after 48 hours in a polyHEMA-coated well compared with mock-infected cells. Viability also appeared enhanced after 24 hours when measured by calcein-AM or after 48 hours when measured with WST-1. Then, we examined whether the down-regulation of HMW-TPM1 isoforms induced by miR-K2 and miR-K5 could inhibit anoikis. To determine that the effect of miR-K2 and miR-K5 on cell viability was plausibly due to the repression of Tmskα1 and/or TM3, we designed siRNAs directed against exon-15 (si-Exon15) and against exon-12 of the TPM1 gene. Again, quantitative western blots confirmed that miR-K2 down-regulated the expression of Tmskα1 and TM3, whereas miR-K5 down-regulated expression of Tmskα1 (Fig 5B and 5C). As expected, si-Exon15 decreased the protein levels of TM3 and TM5 because exon-15 encodes the 3’UTR of these mRNAs. Since exon-12 encodes the C-terminal part of Tmskα1, the siRNA directed against exon-12 reduced the protein level of Tmskα1. Unexpectedly, si-Exon12 also down-regulated TM3 protein levels. The miRNA target prediction program, miRanda, predicted a binding site for si-Exon12 in the exon-3 of TPM1, suggesting that si-Exon12 may also target TM3 mRNA using imperfect complementarity. Exon-3 is shared between the mRNA of Tmskα1 and TM3 but is not present in the mRNA of TM5, which could explain the western blot pattern observed with si-Exon12. Consequently, by targeting both HMW-TPM1 isoforms expressed in HUVECs like miR-K2 and KSHV *de novo* infection, si-Exon12 is a valuable tool to mimic the effect of miR-K2 and KSHV infection on TPM1 isoforms in endothelial cells. Cells knocked-down for Tmskα1 and TM3 by miR-K2 showed a two-fold improved survival rate on polyHEMA plates, compared to control cells transfected with miR-Neg (Fig 5D). In contrast, cells transfected with miR-K5 did not have a significant impact on cell viability, suggesting that TM3 was the isoform of TPM1 involved in anoikis phenotype. Interestingly, si-Exon12 and si-Exon15 also increased viability of endothelial cells after 48 hours of culture on a polyHEMA plate. As mentioned above, those siRNAs down-regulate TM3. These data highlight the importance of TM3 repression by miR-K2 in the inhibition of anoikis.

### KSHV miRNAs enhance tube formation in endothelial cells

**Fig 6:**
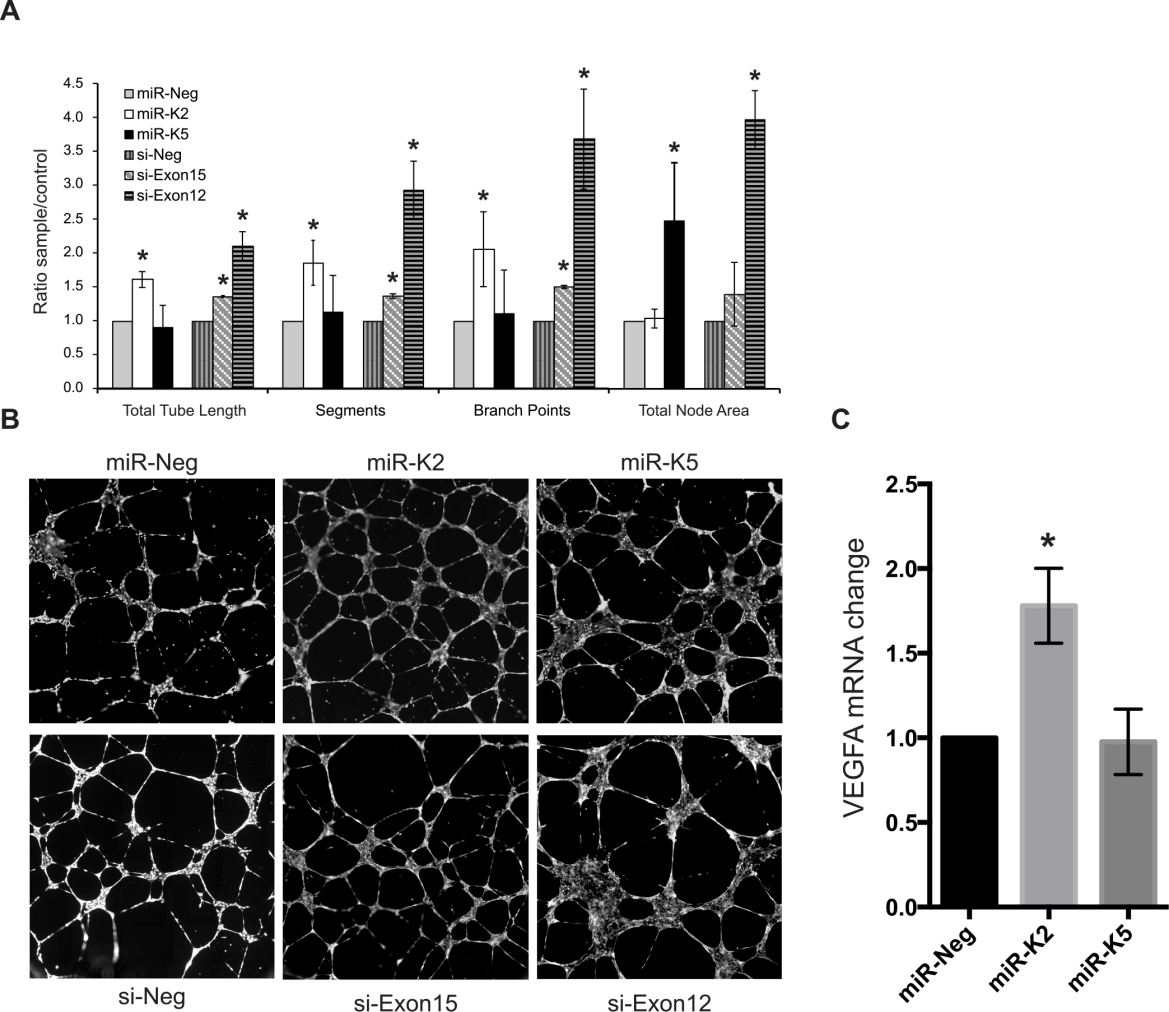
Down-regulation of HMW-TPM1 by miR-K2 and miR-K5 enhances tube formation in HUVECs. Tube formation assays were performed with HUVEC transfected by the indicated RNA. At 36 hpt, HUVECs were seeded on basement membrane matrix extract for 12 hours and stained using calcein AM. The entire well surface was imaged. Whole well images were analyzed using the angiogenesis tube formation module in MetaMorph 7.7 software **(A)** MetaMorph quantification of total tube length (total microns of vessel length excluding nodes), segments (total number of vessel segments connecting branch points and/or ends), branch points (total number of junctions connecting segments, nodes are not considered branches) and total node area (total square microns of connected junctions) formed by HUVECs transfected with the indicated RNA. Average and SD were calculated from four independent experiments. Statistically significant data (p<0.05) are indicated with an asterisk. **(B)** Random fields-of-view of HUVECs grown on basement membrane matrix (field presented here cover approximately 50% of the whole well imaged). (C) HUVECs were transfected with miRNAs and VEGF-A mRNA was measured by RT-qPCR from three independent experiments.

Several reports impute an anti-angiogenic activity to the HMW-forms of TPM1 and TPM2 [30, 31, 33, 53] and KSHV infection enhances tube formation in cell culture conditions [8]. Consequently, we speculated that the reduced expression of HMW-TPM1 induced by miR-K2 and miR-K5 could enhance angiogenesis. Therefore, we performed basement membrane matrix extract tube formation assays with HUVECs previously transfected with miR-K2 or miR-K5 or with siRNAs targeting exon 15 or exon 12 (si-Exon15, si-Exon12). After transfection of miR-K2 mimics, HUVECs formed longer tubes (total microns of vessel length excluding nodes), more segments (total number of vessel segments connecting branch points and/or ends), and more branch points (total number of junctions connecting segments, nodes are not considered branches) compared to control cells (Fig 6A). Similar observations were made with cells transfected by si-Exon15. Because miR-K2 and si-Exon15 both repress TM3, it is possible that the down-regulation of TM3 enhances tube formation. Transfection of miR-K5 mimics in HUVECs did not lead to an increase in tube length, segment or branch points, but HUVECs developed nodes (tube junctions) more than two times larger compared with those developed by cells transfected with a miRNA control (Fig 6B). TM3 and Tmskα1 protein levels were reduced in HUVECs transfected with si-Exon12. Remarkably, these cells formed longer tube structures, with more segments and branch points as cells knocked-down for TM3 by miR-K2 does and developed large nodes as cells knocked-down for Tmskα1 by miR-K5 does. Hence, repression of HMW-TPM1 isoforms by miR-K2 and miR-K5 correlates with tube formation ability of endothelial cells, but it remains possible that other direct and indirect targets (in addition to TPM1) of miR-K2 and miR-K5 contribute to the observed phenotypes. Another marker of angiogenesis is increased expression of VEGFA, which was observed in the presence of miR-K2 (Fig 6C). Together, these data suggest that KSHV miRNAs contribute to an angiogenic environment.

## Discussion

Over the past decade, microRNAs have emerged as important and commonly used regulators of gene expression. Here we report that KSHV, the causative agent of Kaposi's sarcoma, uses microRNAs to down-regulate the HMW-forms of TPM1. Indeed, quickly after infection, Tmskα1 and TM3 were repressed, whereas TM5 decreased slightly only several days after infection. In HUVECs, miR-K2 repressed the protein level of Tmskα1 and TM3 likely through targeting a sequence within the coding region of these transcripts. Furthermore, inhibition of miR-K2 with LNA during KSHV infection increased expression of Tmskα1 and TM3. KSHV miR-K2 decreased anchorage-dependent cell death and stimulated angiogenesis of endothelial cells. Additionally, we identified a binding site for miR-K5 in the 3’UTR of Tmskα1 mRNA, and confirmed that miR-K5 reduced the expression level of Tmskα1 in HUVECs. The correlation of phenotypes associated with KSHV miRNAs and siRNAs specifically targeting TPM1 also correlate with repression of TPM1. However, it remains possible that the phenotypes observed with the KSHV miRNAs are the result of repression of TPM1 and other host factors since miRNAs are known to target many genes simultaneously.

In HUVECs, we detected TM5, an LMW-TPM1 isoform not previously described in these cells. KSHV miR-K10a reduces expression of a luciferase reporter carrying exon-15. Additionally, transfection of miR-K10a represses TM5 in HUVEC. However, repression of TM5 does not appear to be explained by a direct binding of miR-K10a on the 3’UTR of TM5 mRNA because TM5 and TM3 mRNA share the same 3’UTR and the protein level of TM3 was not affected by miR-K10a. It is unclear at this time how miR-K10a repressed the luciferase activity of the reporter carrying exon-15 and did not decrease the protein levels of TM3 in HUVECs. During *de novo* infection, repression of TM5 is observed several days after down-regulation of Tmskα1 and TM3. Given these findings, it is unlikely that expression of TM5 is regulated by KSHV miRNAs in infected cells.

KSHV miRNAs inhibit apoptosis though multiple ways [12]. For instance, KSHV miRNAs repress the expression level of pro-apoptotic factors such as caspase 3 [54]. KSHV miRNAs also inhibit expression of the receptor of the TNF-related weak inducer of apoptosis (TWEAK) [38]. We discovered that KSHV miRNAs block another apoptotic pathway named anoikis. To survive, endothelial cells need to be anchored to a matrix. Qian et al. [55] reported that in HUVECs, KSHV infection induces secretion of metalloproteinase. The metalloproteinases secreted by infected endothelial cells degrade the extra-cellular matrix, and release cells from their natural anchorage. In addition, KSHV K5 protein down-regulates VE-cadherin [56]. Consequently, adherens junctions between cells are disrupted and invasiveness of KSHV-infected cells is facilitated. Free of their contacts, endothelial cells undergo anoikis, but ectopic expression of the KSHV protein vFLIP diminished this effect. Indeed, dermal microvascular endothelial cells expressing vFLIP survive better than control cells when cultured on polyHEMA [52]. Furthermore, in breast cancer cells repression of HMW-TPM1 inhibit anoikis whereas over-expression of HMW-TPM1 suppresses transformed phenotype and anchorage independent growth [25, 26, 57]. In cancer cells, down-regulation of HMW-TPM1 expression could be the result of activation of the Ras/Raf/MEK/ERK pathway [58], or DNA methylation of TPM1 promoter [59], or over-expression of the onco-microR-21 [27]. So far, the mechanism by which the reduction of HMW-TPM1 expression inhibits anoikis remains unknown. In this study, we found that KSHV infection inhibits anoikis by an additional mechanism. Consistent with results from breast cancer models, down-regulation of the TPM1 isoform TM3 by miR-K2 or by a siRNA enhances viability of cells that have become detached from a growth surface. Together, our results highlight the importance of KSHV miRNA in the dissemination process of KSHV-infected endothelial cells. Interestingly, in gastric carcinoma cells, miRNAs expressed by Epstein-Barr virus repress TPM1 and induce anchorage-independent growth [60] suggesting that down-regulation of TPM1 by viral-miRNAs could be a general mechanism used by herpesviruses to inhibit anoikis.

Multiple miRNA targets of KSHV are associated with vascularization. Indeed, the first KSHV miRNA target described was the anti-angiogenic factor thrombospondin 1 [61]. More recently it has been reported that KSHV reduces the protein expression of another anti-angiogenic factor, Delta-like 4 (DLL4), by promoting the expression of specific host miRNAs [62]. Here we found that KSHV miRNAs stimulate angiogenesis by repressing the protein level of HMW-TPM1. It has been proposed that HMW-forms of TPM1 are exported to the cell surface and utilized as receptors for anti-angiogenic ligands like HKa, HPRG and endostatin [34]. These conclusions are supported by the loss of the anti-angiogenic activity of HKa, HPRG and endostatin on cells treated with the antibody TM311, which recognizes the N-terminal part of the HMW-forms of TPM1 and TPM2 [63]. Interestingly, TM311 was used to show that endostatin interacts with TPM filaments in the cytosol of KS cells [32]. In our study, we demonstrated that down-regulation of TM3 by miR-K2 enhances tube formation of HUVECs. It is likely that down-regulation of intracellular TPM1 by miR-K2 decreases the amount of TPM1 molecules at the cell surface. In fact, HUVECs were not treated with additional plasma ligands in our assays. Therefore, the pro-angiogenic effects of miR-K2 do not appear to be due to an inhibition of the transduction of an exogenous extracellular signal.

The TPM proteins are a vast family of actin binding protein encoded by four different genes and all four genes generate multiple isoforms. The functions of TPM isoforms are poorly understood. Here we have demonstrated that suppression of specific TPM1 isoforms Tmskα1 and TM3 by KSHV miRNAs miR-K2 and miR-K5 have unique functional effects in the context of KSHV-infected endothelial neoplasms. Indeed, down-regulation of TM3 by miR-K2 increases anchorage-independent growth and may facilitate KS dissemination. Furthermore, mRNA expression profiling revealed that products of TPM2 and TPM4 genes were down-regulated during KSHV infection, whereas TPM3 expression is unchanged (Fig 1B). It seems that KSHV induces a drastic remodeling of the actin filaments [64]. In all likelihood, suppression of TM3 and Tmskα1 leads to their replacement on the actin filament by other TPM proteins. Through repression of HMW-TPM1 isoforms by the miRNAs, less HMW-TPM1 proteins would be able to interact with actin filaments. This could in turn alter the association of various proteins with actin, like myosin II and other TPM isoforms. This would not necessarily alter the actin cytoskeleton by changing the ratio of filamentous to globular actin, but could change the elasticity of F-actin networks and the organization of actin bundles [65]. HMW-TPM inhibition also correlated with decreased lamellipodia and protrusion persistence [66]. These changes in cell stiffness and protrusion might benefit increased tube formation in endothelial cells. Such a redistribution of the TPM population on actin filaments by KSHV likely changes the dynamic properties of the cytoskeleton. Consequently, the re-composition of actin filaments in KSHV-infected endothelial cells may explain increased angiogenesis of KSHV-infected cells. Infiltrating lymphocytes may detach endothelial cells from other cells or extracellular matrix and trigger anoikis. Our results suggest KSHV miRNAs may prevent apoptosis of KSHV-infected cells when their attachment to adjacent cells and the extracellular matrix is changing in an *in vivo* setting.
